# Endoscopic Ultrasonic Aspiration of Superficial Bladder Tumors

**DOI:** 10.1155/DTE.2.29

**Published:** 1995

**Authors:** Makoto Miki, Hiroaki Shiozawa

**Affiliations:** Department of Urology Tokyo Medical College 6-7-1 Nishishinjuku, Shinjuku-ku Tokyo 160 Japan

## Abstract

The gold standard of surgical treatment for superficial bladder tumor is transurethral resection (TUR). However, the procedure has drawbacks: it needs skill to avoid perforation of the bladder wall, while burns on the resected tissue are disadvantageous for histological examination. We developed an endoscopic ultrasonic aspiration system which is easy to manipulate and makes no burns on aspirated tissue. Furthermore, the equipment includes a coagulating system.

Transurethral ultrasonic aspiration was performed in 19 cases of superficial bladder tumor with a prototype 24Fr Y-shaped rigid endoscope. The efficacy of bladder tumor aspiration was evaluated and ultrasonic aspiration was compared to the conventional TUR procedure. Bladder tumors less than 2 cm were aspirated very rapidly. Blood loss was minimal and the largest tumor aspirated in this series weighed 6.5 grams. Aspirated tissue was large enough for histological examination. Compared to conventional TUR, the ultrasonic aspiration has advantages of no burns on aspirated tissue and easy manipulation. Transurethral ultrasonic aspiration may be an alternative procedure for the treatment of superficial bladder tumor.


*Diagnostic and Therapeutic Endoscopy,* 1995, Vol. 2, pp. 29–33
Reprints available directly from the publisher
Photocopying permitted by license only

© 1995 Harwood Academic Publishers GmbH
Printed in Singapore

### Endoscopic Ultrasonic Aspiration of Superficial Bladder Tumors

MAKOTO MIKI and HIROAKI SHIOZAWA

*Department of Urology, Tokyo Medical College, Nishishinjuku, Shinjuku-ku, Tokyo, Japan 160.*

*(Received January 20, 1995; in final form March 10, 1995)*

The gold standard of surgical treatment for superficial bladder tumor is transurethral resection (TUR). However, the procedure has drawbacks: it needs skill to avoid perforation of the bladder wall, while burns on the resected tissue are disadvantageous for histological examination. We developed an endoscopic ultrasonic aspiration system which is easy to manipulate and makes no burns on aspirated tissue. Furthermore, the equipment includes a coagulating system.
Transurethral ultrasonic aspiration was performed in 19 cases of superficial bladder tumor with a prototype 24Fr Y-shaped rigid endoscope. The efficacy of bladder tumor aspiration was evaluated and ultrasonic aspiration was compared to the conventional TUR procedure. Bladder tumors less than 2 cm were aspirated very rapidly. Blood loss was minimal and the largest tumor aspirated in this series weighed 6.5 grams. Aspirated tissue was large enough for histological examination. Compared to conventional TUR, the ultrasonic aspiration has advantages of no burns on aspirated tissue and easy manipulation. Transurethral ultrasonic aspiration may be an alternative procedure for the treatment of superficial bladder tumor.

**KEY WORDS:** Bladder tumor, transurethral surgery, ultrasonic aspiration

### INTRODUCTION

Surgical treatment for superficial bladder tumors has generally been performed by TUR (1–3). TUR is a minimally invasive and convenient method, however, the procedure needs skill to avoid perforating the bladder wall during resection of the base of the tumor. Moreover, electrical burns occur on the resected tissue which hinders histological examination. We developed an endoscopic ultrasonic aspiration system (4) which is easy to manipulate and does not burn the aspirated tissue. Transurethral ultrasonic aspiration of superficial bladder tumor was performed with this equipment, and the efficacy was evaluated in comparison to conventional TUR.

Address for correspondence: Makoto Miki, Department of Urology, Tokyo Medical College, 6-7-1 Nishishinjuku, Shinjuku-ku, Tokyo, Japan 160.

### MATERIALS AND METHODS

Transurethral ultrasonic aspiration was performed in 19 cases of superficial bladder tumor. The ages of patients ranged from 53 to 82 years (17 males and 2 females). Preoperative biopsy revealed that all tumors were transitional cell carcinoma: grade 1 (10), grade 2 (6), grade 3 (3), and they were diagnosed as stage T1 (18) or T2 (1). Transurethral resection was indicated in all cases. The endoscopic ultrasonic aspirator (Fig. 1) used in this series was developed in cooperation with Olympus Optical Co. Ltd. and is equipped with a coagulation system. Two types of titanium probes were used, one with a straight distal tip and the other with a tapered distal tip (Fig. 2). They were long enough for use through a channel of an endoscope and strong enough to allow the use of high amplitude ultrasound (200–300 μm). Both types of probes could be used for coagulation at bleeding sites. For ultrasonic aspiration, a special prototype 24 Fr Y-shape rigid endo-

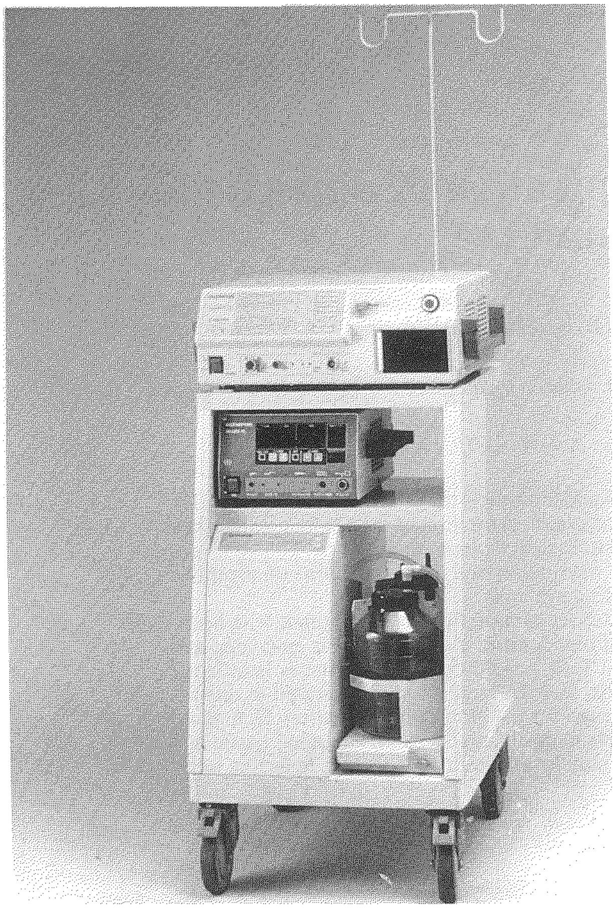

**Figure 1** The generator of the endoscopic ultrasonic aspiration system (external dimensions 480 x 660 x 1070 mm) equipped with coagulation system (Surgical unit UES-10®).

scope (Fig. 2) was used which was equipped with a stabilizer for the probe and insulated to prevent leakage of coagulating current.

In the preliminary trial, most tumors no larger than 2 cm in diameter were aspirated within 10 minutes. Therefore, after 10 minutes transurethral ultrasonic aspiration of bladder tumor, conventional TUR with the regular sheath and scope was performed to confirm the residual tumor. The amplitude of the sound wave at the tip of the probe in this series could be adjusted from 200 μm to 240 μm. The irrigation volume of sorbitol solution was controlled through the generator to prevent the increase of temperature of the probe in the channel of the scope. Suction pressure was also controlled for appropriate aspiration. A collection trap with a filter was attached to the suction tube to trap the aspirated tissue. During the aspiration, the site of continuous bleeding was coagulated with the same probe. The straight and tapered type probes were used at random and their aspiration characteristics were observed. The aspirated specimen in the collection trap was examined histologically.

### RESULTS

In all cases, tumors were aspirated safely and the largest tumor aspirated in this series weighed 6.5 grams. The tumors smaller than 2 cm in diameter were completely aspirated using only this equipment. However, tumors which were >2 cm and/or T2 were not completely resected with only this equipment. On those occasions conventional TUR was performed. The ultrasound energy had no effect on the obturator nerve during this procedure. It was difficult for the rigid probe to approach tumors on the anterior wall and the neck of the bladder. The specimens aspirated by the straight type probe were sometimes cylindrical in shape and ones aspirated with the tapered type were partially emulsified. The coagulating function using high frequency current through the probe of the ultrasonic aspirator was as effective as that of TUR and loss of blood was minimal. The aspirated tissue specimens could be harvested easily in the separate magazine-chamber type collection trap attached to the suction tube (Fig. 3). For histological examination, specimens aspirated with both the straight type and tapered type probes were large enough for histological examination (Fig. 4), however, one case aspirated with tapered type probe was not suitable for histological examination because of emulsification. None of the aspirated specimens in any of the 19 cases were disfigured by electrical burns.

### DISCUSSION

In order to enable easier and safer urological endoscopic surgery, we have developed an endoscopic ultrasonic aspiration system with a coagulating function. Krawitt and Addonizio reported effective transurethral ultrasonic aspiration for the prostate as well as stones (5). However, other reports suggested that the applications of ultrasonic aspirators in endoscopic surgery were limited to stones (amplitude: 50–80 μm), because conventional probes long enough for endoscopic surgery are too fragile to allow the use of high amplitude ultrasound which can aspirate various tissues (amplitude: 200–300 μm). Therefore, we developed a new ultrasonic aspiration system equipped with long and strong titanium probes for endoscopic surgery. In this study, this equipment was evaluated in transurethral surgery of superficial bladder tumor.

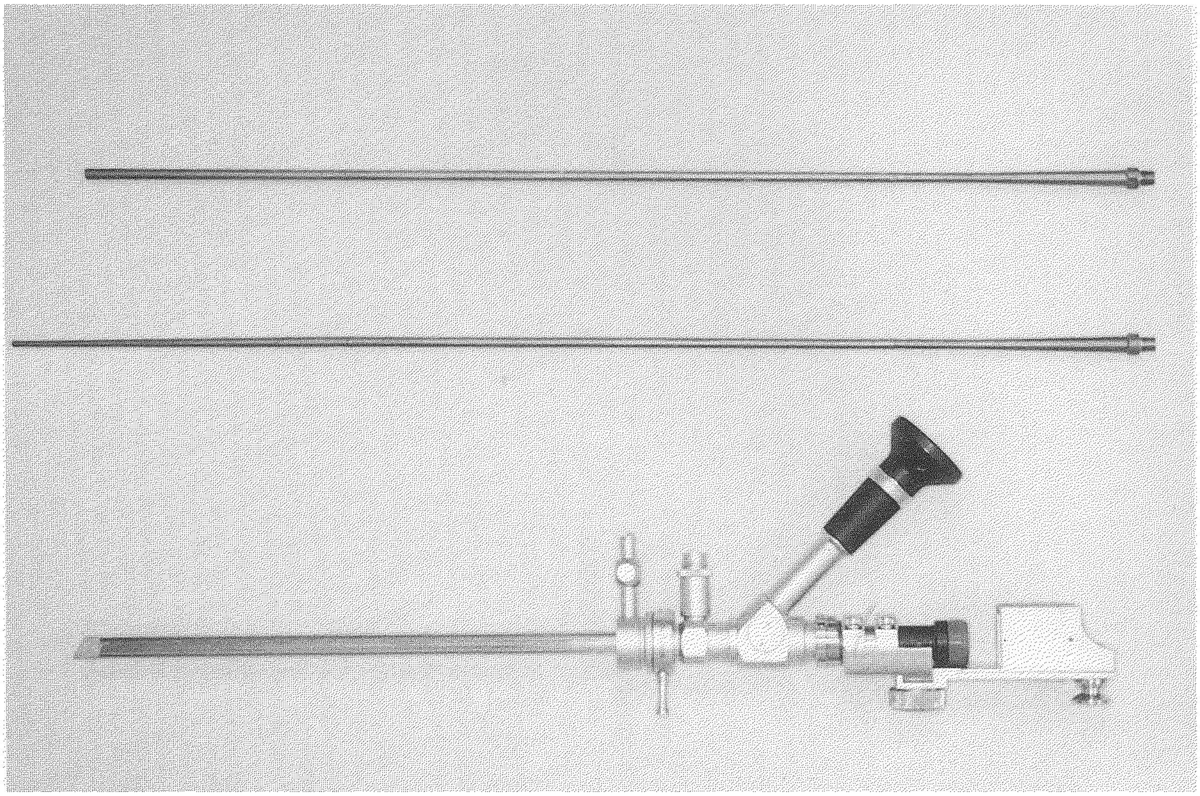

**Figure 2** Two types of probes: straight type (upper) and tapered type (middle) and the 24 Fr endoscope equipped with the stabilizer (lower).

Superficial bladder tumors are generally treated by transurethral surgery. Transurethral resection has been the gold standard for the treatment of superficial bladder tumors and minimally invasive surgery, compared to open surgery. However, there are reports that the rate of perforation of the bladder wall during TUR for bladder tumor was 3 to 5% (2,6). In our experience between 1990 and 1994, only 2 cases (1%) suffered perforation out of 203 cases of TUR for bladder tumor. Although TUR is performed widely and the rate of perforation is low, the procedure needs skill in order to prevent perforation of the bladder wall during resection of the muscle layer. In this series, ultrasonic aspiration of the base of the tumor was not performed because of inefficient aspiration of the muscle layer. By changing the shape of the tip of the probe to a scalpel-like tip, it might be possible to aspirate the base of the tumor more safely than TUR which uses electrocautery. The ultrasound energy had no effect on the obturator nerve in the bladder, however we recognized stimulation of the obturator nerve when the tip of the probe touched it directly during laparoscopic lymphadenectomy in another series of cases.

With TUR, electrical burns are usually inflicted on the specimen. The burns sometimes make histological diagnosis difficult, especially in small tumors. The aspirated specimens obtained by the ultrasonic aspiration system had no burns, which contributed to accurate histological diagnosis. The difference of characteristics between the straight type and tapered type probe appeared in aspirated specimen. The specimens aspirated by the straight type probe were all large enough to establish a histological diagnosis and sometimes were cylindrical in shape. The specimens aspirated by the tapered type probe were emulsified and in one case, histological diagnosis was impossible because of emulsification of the small aspirated specimen.

The implantation of tumor cells, which is possible during TUR (7,8) must also be considered with this method. While theoretically, aspiration should minimize the scattering of tumor cells during the procedure, mucosa surrounding the tumor occasionally became edematous during aspiration with the straight type probe, therefore this phenomenon needs more examination. All tumors smaller than 2 cm could be aspirated completely within

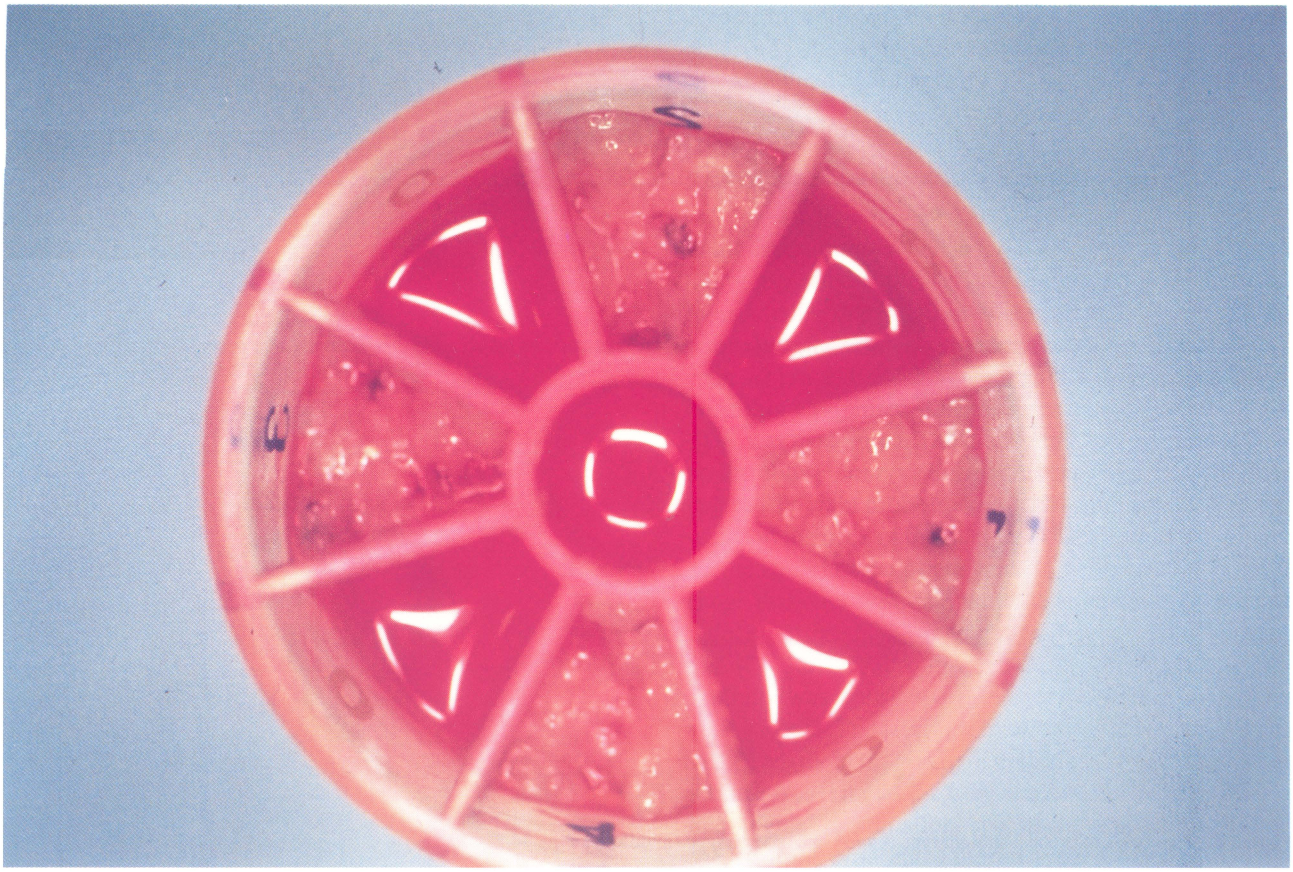

**Figure 3** The tumor specimen collected in the container is slightly emulsified.

10 minutes. For such small tumors, ultrasonic aspiration was as valuable as conventional TUR.

In this series, some problems of transurethral ultrasonic aspiration of bladder tumor were recognized such as 1) difficulty of distinction between Ta and T1 because of the small size of specimen, 2) inefficient aspiration of the muscle layer including the base of the tumor, 3) difficulty for a rigid probe to approach tumors on the anterior wall and neck of the bladder. In such cases conventional TUR was needed. A scalpel-like tip could be used to obtain a large enough specimen to distinguish Ta and T1 and scoop out the muscle layer. Furthermore, a scalpel attached to equipped on the side of the tip would make it possible to scoop out tumors on the anterior wall and the neck of the bladder without having to place the tip at 90° to the tumor.

Transurethral ultrasonic aspiration of superficial bladder tumor is safe and the equipment is easier to manipulate than TUR. Furthermore, the absence of burns on the aspirated tissue is an advantage compared to TUR. Although this system does not have any cost benefit if it is used only for bladder tumors, the ultrasonic aspirator equipped with a coagulation function can be also used in laparoscopic surgery. We evaluated the efficacy of transurethral ultrasonic aspiration for bladder tumors in comparison to TUR. While the procedure is not yet as effective clinically as conventional TUR, a control study with TUR is needed for transurethral ultrasonic aspiration to be accepted as one clinical method for the treatment of bladder tumors in the future.

### ACKNOWLEDGMENTS

The authors are grateful to Olympus Optical Co., Ltd. for their assistance and cooperation in developing this equipment. They are also grateful to Professor J. Patrick Barron of Tokyo Medical College for his review of this English manuscript.

This study was assisted by a Grant-in-Aid for General Scientific Research (B) of the Ministry of Education of Japan.

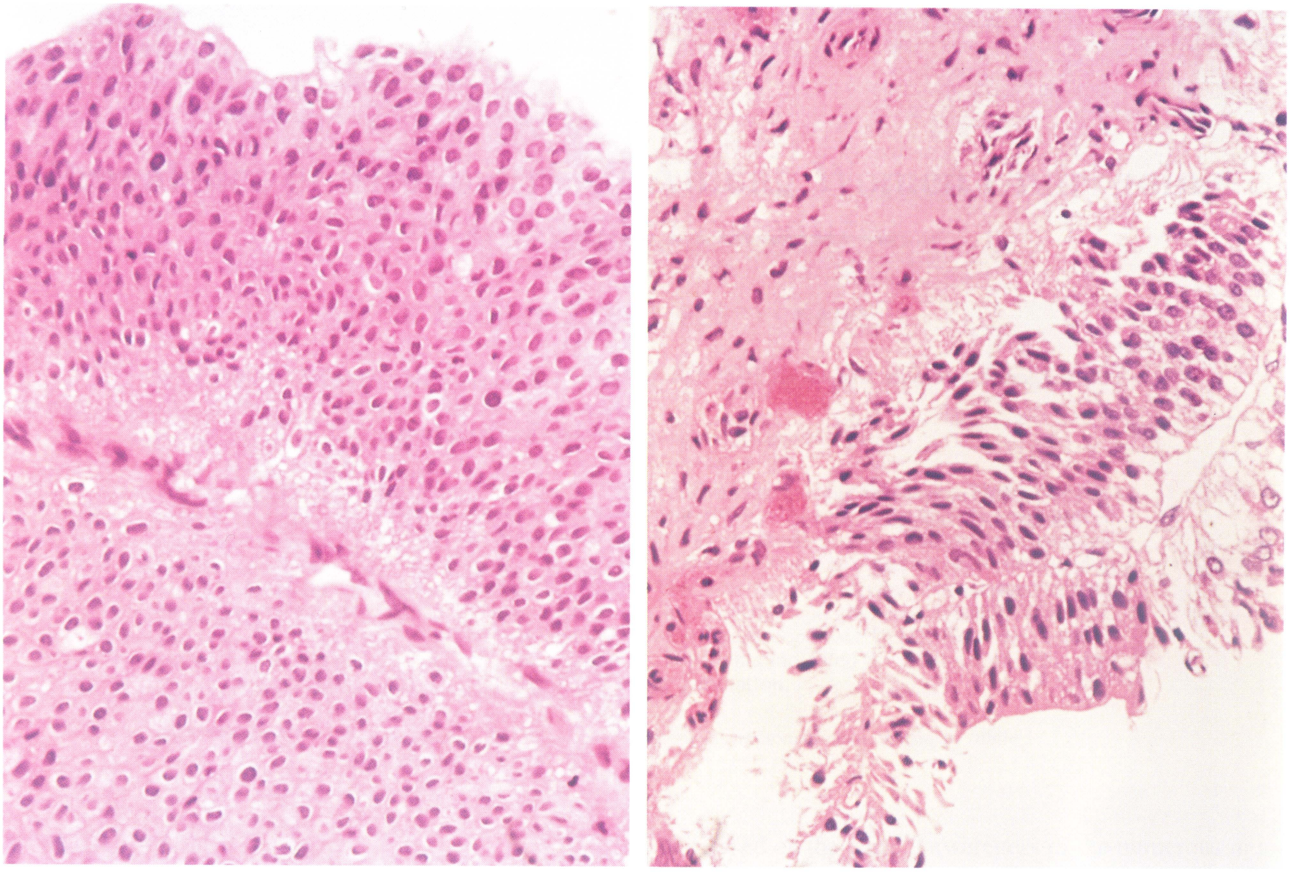

**Figure 4** Histological findings: tumor (transitional cell carcinoma grade 2) aspirated by the straight type probe (left) and tapered type probe (right). Both specimens have no burns or eschar although the latter is slightly distorted.

### REFERENCES

1. Jewett HJ. Carcinoma of the bladder: diagnostic appraisal and choice of treatment. J. Urol. 1961;86:572–582.
2. Koshiba K. Transurethral resection for carcinoma of the urinary bladder. Jpn. J. Urol. 1964;55:843–868.
3. Barnes RW, Bergman RT, Hadley HL, et al. Control of bladder tumors by endoscopic surgery. J. Urol. 1967;97:864–868.
4. Shiozawa H, Miki M. A newly developed endoscopic ultrasonic aspiration system and its clinical application. Minimal. Invasive Ther. 1993;2:261–264.
5. Krawitt DR, Addonizio JC. Ultrasonic aspiration of prostate, bladder tumors and stones. progress report. J. Urol. 1987;30:579–580.
6. Dick A, Barnes R, Hadley H, et al. Complications of transurethral resection of bladder tumors: prevention, recognition and treatment. J. Urol. 1980;124:810–811.
7. Boyd PJR, Burnand KG. Site of bladder-tumour recurrence. Lancet 1974;2:1290–1292.
8. Page BH, Levison VB, Curwen MP. The site of recurrence of non-infiltrating bladder tumours. Brit. J. Urol. 1978;50: 237–242.

